# Editorial: Positive leadership and worker well-being in dynamic regional contexts

**DOI:** 10.3389/fpsyg.2023.1349522

**Published:** 2024-01-08

**Authors:** Martijn Burger, Martine J. H. Coun, Jol Stoffers, Steven Van Den Heuvel, Arne Vanderstukken, Thomas Van Waeyenberg

**Affiliations:** ^1^Department of Organisation, Open Universiteit, Heerlen, Netherlands; ^2^Erasmus Happiness Economics Research Organisation (EHERO), Voor Erasmus University, Rotterdam, Netherlands; ^3^School of Economics, University of Johannesburg, Johannesburg, South Africa; ^4^Research Centre for Employability, Zuyd University of Applied Sciences, Sittard, Netherlands; ^5^Research Centre for Education and the Labour Market (ROA), Maastricht University, Maastricht, Netherlands; ^6^Department of Systematic Theology, Evangelical Theological Faculty, Leuven, Belgium

**Keywords:** positive leadership, worker, employees, wellbeing, dynamic contexts

## Introduction

In this Research Topic, we present a collection of research articles that explore the interplay between positive leadership styles and worker wellbeing in dynamic regional contexts. In the literature, it has been well-established that leaders have an effect on employee performance and worker wellbeing (i.e., the general wellbeing of working people; Arnold, 2017; Wijngaards et al., 2022) within organizations (e.g., Kuoppala et al., 2008; Montano et al., 2017; Hendriks et al., 2020) and that destructive leadership is detrimental for worker wellbeing (Schyns and Schilling, 2013; Mackey et al., 2021). At the same time, the relationship between positive leadership styles and worker wellbeing is significantly influenced by the unique characteristics and dynamics of the context in which an organization is located through cultural norms in a country, industry trends, economic conditions, and demographic patterns (Stoffers, 2021, 2023).

Specifically, in this Research Topic several studies explore the relationship between positive leadership styles (e.g., empowering, health-related, transformational, self-sacrificial, servant, service, and virtuous leadership) and worker wellbeing. The included articles demonstrate that worker wellbeing can be operationalized in various ways, including life satisfaction, job satisfaction, and employee vigor. Some articles additionally examine more individual and organizational performance-related outcomes such as innovative behavior, employability and turnover intention. The diversity of articles in this Research Topic illustrates that positive leadership styles that work in one context (e.g., region or sector) does not necessarily work in other contexts. Hence, taking contextual factors into account—through case studies –, allows for a better understanding of *what works for whom under which circumstances* (Nielsen and Miraglia, 2017).

By using an interdisciplinary approach which incorporates insights from organizational psychology, organizational sociology, management sciences, human resource management, and labor economics, this Research Topic contributes to a better comprehension of the role of leadership and context in promoting worker wellbeing. We believe that the findings from this Research Topic can enhance the development of more effective, adaptable, and context-sensitive leadership approaches, ultimately fostering worker wellbeing and promoting the flourishing of industries, regions and organizations. Furthermore, these insights can help shape future organizations and societies that prioritize worker wellbeing, leading to increased job satisfaction, engagement, and overall quality of life. We express our gratitude to all the contributing authors and reviewers, whose diligent efforts have shaped and refined the articles in this Research Topic.

## In this Research Topic

The majority of the articles focus merely on one specific context concerning three themes: (1) conceptualization and measurement of positive leadership behavior and (2) the relationship between positive leadership and worker wellbeing, and (3) the relationship between positive leadership and individual and firm performance.

Three articles offer distinct perspectives on leadership behaviors and their conceptualization and measurement within organizational settings, each contributing unique insights to the overarching theme of positive leadership behavior.

The research of Slob et al. delves into Augustinian leadership principles, focusing on the centrality of community within organizations. The proposed Augustine leadership scale is notable for incorporating ethical dimensions such as veracity and empathy, alongside community orientation and temperance. This approach suggests that leadership success is rooted not only in organizational outcomes but also in the cultivation of a strong, value-driven community.

The work of Nöthel et al. introduces the Adaptive Leadership Behavior Scale (ALBS), a novel metric for gauging adaptive leadership behaviors, crucial for navigating dynamic and challenging environments. This scale measures leaders' abilities to perceive situational nuances, possess a versatile repertoire of behavioral strategies, and apply these behaviors flexibly and judiciously. The validation of the ALBS furthers our understanding of how leaders can effectively adjust to and manage change.

Henderikx and Stoffers' study shifts the focus to middle managers, particularly in the context of digital transformation—a radical and pervasive change affecting modern organizations. Through Group Concept Mapping, they identify crucial leadership behaviors and skills, emphasizing the significance of soft skills and people-oriented behaviors for successfully leading digital transformation efforts.

Connecting these articles, a comprehensive picture emerges, portraying leadership as multifaced behavior that thrives on ethical grounding (Augustinian leadership), adaptability to change (Adaptive Leadership Behavior), and a keen understanding of the human element in technological advancements (Middle-Managers' Leadership Skills). Together, they imply that positive leadership behavior is not a static trait but a dynamic set of skills and orientations that must evolve with organizational challenges and changes. This synthesis highlights the complexity of leadership and underscores the need for diverse and robust conceptualizations and measures to capture the essence of effective leadership across different contexts.

The subsequent five articles collectively explore the intricate relationship between various leadership styles and worker wellbeing, each contributing unique insights that, when connected, offer a multi-dimensional view of leadership effectiveness in diverse contexts.

Habets et al. set the stage with a cross-cultural examination of dark leadership in the Netherlands and China, observing its negative impact on Dutch workers' turnover intentions when moderated by a supportive learning climate. In contrast, this effect was not observed in the Chinese context, suggesting that cultural factors significantly influence how leadership styles affect employee outcomes.

Caniëls work extends the conversation to remote work environments, highlighting how positive leadership becomes even more crucial for employee vigor in the absence of traditional office interactions. Studied within the Netherlands and Flanders, the findings suggest that remote work amplifies the need for leaders to actively engage and support their teams to maintain productivity and wellbeing.

Cremers and Curşeu contribute to the theme by examining how empowering leadership during the COVID-19 pandemic in the Netherlands can alleviate adverse effects on work satisfaction and team effectiveness. Their research underscores the importance of leaders who facilitate autonomy and confidence among employees during crises.

Shek et al. explore the concept of self-leadership within the context of service leadership among Hong Kong university students. The study showcases self-leadership's positive correlation with wellbeing, indicating that the ability to self-direct and self-motivate is beneficial for psychological health in young adults.

Lastly, Stynen and Semeijn investigate the role of paradoxical leadership—a style that embraces contradictory behaviors for adaptability—on employee wellbeing during turbulent times. Their findings suggest that paradoxical leadership can enhance job, career, and life satisfaction by helping employees navigate and reframe challenging work conditions.

Connecting these articles, it's evident that leadership has a profound and varied impact on worker wellbeing across different cultural and situational contexts. From the detrimental effects of dark leadership to the empowering aspects of positive and paradoxical leadership, each study contributes to a broader understanding of how leaders can support or undermine employee wellbeing. The common thread is the significance of adapting leadership styles to the cultural, situational, and individual needs of workers to foster an environment conducive to satisfaction, effectiveness, and overall wellbeing.

The final four articles form a cohesive narrative around the theme of positive leadership's impact on individual and organizational performance, with a focus on employability, creativity, job satisfaction, and the mechanisms through which leadership exerts its influence.

Vermeeren and Van der Heijden delve into the public sector's employability, underscoring the interaction between individual traits like personality and risk-taking, and organizational features such as transformational leadership. Their study indicates that leadership styles interwoven with personal and institutional factors critically affect employees' career development and labor market agility.

Cao et al. pivot to explore the effect of leader humor on employee creativity in China, revealing that humor enhances creativity and is mediated by employees' willingness to voice ideas and concerns. They further elucidate the moderating role of contradiction thinking, which amplifies the impact of humor on more radical forms of creativity, with variations noted between state-owned and private sectors.

Coun et al. investigate the remote work landscape, examining how servant leadership and communication frequency across various channels contribute to job satisfaction among different generations. Their findings affirm that servant leadership and regular communication, particularly via email, are perceived as autonomy-supportive and positively influence employees' wellbeing across generational divides.

Hoedemakers et al. present a bibliometric analysis and systematic literature review probing how leadership influences employability. They underscore the dyadic relationship quality between leaders and followers as pivotal, as it determines leaders' propensity to provide resources such as training and feedback, thereby enhancing employability. The article posits that cultivating leadership skills is a strategic HRM investment that bolsters employability, offering a roadmap for future research and practical applications.

The common thread through these articles is that positive leadership behaviors—whether through humor, transformational practices, servant leadership, or effective communication—are fundamental drivers of employee and organizational outcomes. Leadership not only impacts immediate performance but also equips employees with the skills and motivation necessary for long-term employability and adaptability. These studies highlight then also the importance of understanding the nuances of leadership across cultural and generational contexts to optimize both individual growth and firm performance.

In sum, the studies in this Research Topic enhance our understanding of the interplay between leadership styles, worker wellbeing and worker performance. By considering context characteristics and dynamics, this research contributes to the development of more effective and adaptable leadership approaches that prioritize worker wellbeing, paving the way for thriving organizations and societies in an ever-evolving global economy. We invite you to explore the diverse range of topics and ideas presented in this Research Topic and engage with the authors and fellow readers to stimulate dialogue and mutual learning.

## Future research

In this Research Topic, we showed that positive leadership behavior can play an important role in wellbeing of workers. Nevertheless, literature on the role of leadership for leader's own wellbeing and vice versa the interaction with employee's wellbeing is scarce (Inceoglu et al., 2018, 2021). Future research could address this gap by focusing on which factors influence the wellbeing of leaders and to what extent the behavior of leaders at one level (e.g., middle managers) influence the behavior and wellbeing of leaders at lower levels and to what extent does this in turn influence the wellbeing of the employees (see e.g., Lin et al., 2019).

## Author contributions

MB: Writing—review & editing. MC: Writing—review & editing. JS: Writing—review & editing. SV: Writing—review & editing. AV: Writing—review & editing. TV: Writing—review & editing.
